# A Comparative Study on the Detection of Covert Attention in Event-Related EEG and MEG Signals to Control a BCI

**DOI:** 10.3389/fnins.2017.00575

**Published:** 2017-10-16

**Authors:** Christoph Reichert, Stefan Dürschmid, Hans-Jochen Heinze, Hermann Hinrichs

**Affiliations:** ^1^Department of Behavioral Neurology, Leibniz Institute for Neurobiology, Magdeburg, Germany; ^2^Department of Neurology, Otto-von-Guericke University, Magdeburg, Germany; ^3^German Center for Neurodegenerative Diseases (DZNE), Magdeburg, Germany; ^4^Center for Behavioral Brain Sciences, Magdeburg, Germany

**Keywords:** multi-modal control, brain-computer interface, spatial filter, CCA, ERP

## Abstract

In brain-computer interface (BCI) applications the detection of neural processing as revealed by event-related potentials (ERPs) is a frequently used approach to regain communication for people unable to interact through any peripheral muscle control. However, the commonly used electroencephalography (EEG) provides signals of low signal-to-noise ratio, making the systems slow and inaccurate. As an alternative noninvasive recording technique, the magnetoencephalography (MEG) could provide more advantageous electrophysiological signals due to a higher number of sensors and the magnetic fields not being influenced by volume conduction. We investigated whether MEG provides higher accuracy in detecting event-related fields (ERFs) compared to detecting ERPs in simultaneously recorded EEG, both evoked by a covert attention task, and whether a combination of the modalities is advantageous. In our approach, a detection algorithm based on spatial filtering is used to identify ERP/ERF components in a data-driven manner. We found that MEG achieves higher decoding accuracy (DA) compared to EEG and that the combination of both further improves the performance significantly. However, MEG data showed poor performance in cross-subject classification, indicating that the algorithm's ability for transfer learning across subjects is better in EEG. Here we show that BCI control by covert attention is feasible with EEG and MEG using a data-driven spatial filter approach with a clear advantage of the MEG regarding DA but with a better transfer learning in EEG.

## Introduction

In recent years high effort has been made in the development of brain-computer interfaces (BCI). A BCI is intended to recognize voluntary modulated brain signals in order to regain communication and motor control in severely paralyzed patients. The three main types of brain signals applied for BCI control are the P300 potential, often used in so called matrix spellers (Farwell and Donchin, 1988), the μ-rhythm, an oscillation which is suppressed during motor execution and motor imagery (MI) (Wolpaw et al., 1991), and the steady-state visual evoked potential (SSVEP), which reflects oscillatory activity of the visual cortex driven by steady-state visual stimulation (Middendorf et al., 2000). Invasive techniques such as electrocorticography (ECoG) and local field potentials (LFP) provide superior signal quality compared to noninvasive techniques but bear critical shortcomings concerning long-term use and health risks (Schalk and Leuthardt, 2011). Consequently, as a noninvasive technique, electroencephalography (EEG) is commonly used in BCI applications. In contrast, the magnetoencephalography (MEG) as another noninvasive technique has a better spatiotemporal resolution (Hämäläinen et al., 1993) but bears practical limitations for BCI use. It is considered an orthogonal complement of EEG, which provides additional value. Specifically, in the field of brain imaging MEG has been shown to provide better spatial resolution in source reconstruction compared to concurrent EEG (Leahy et al., 1998) and to be superior in detecting deep coherent sources during voluntary movements (Muthuraman et al., 2014). In contrast, others claim that there is no difference in the accuracy of both methods (Liu et al., 2002; Baumgartner, 2004). However, in a recently published review article (Baillet, 2017) the authors emphasize the strength of MEG over EEG.

Despite the obvious advantages of MEG, it has rarely been used for BCI development, certainly due to its limited practical applicability. Nevertheless, the capabilities of MEG should be exploited for BCI algorithm development and as potential application for rehabilitation and patient training. So far, several attempts have been made in the field of MEG based BCIs. For example, Buch et al. (2008) have trained stroke patients in multiple sessions to control a BCI by MI. Spüler et al. (2014a) have found coherence discriminating mental tasks in an MEG BCI. Furthermore, Florin et al. (2014) demonstrated real-time MEG application in the source space by neurofeedback targeting to increase the theta-to-alpha ratio. While these studies demonstrate the suitability of MEG for BCIs, the direct comparison with EEG based BCIs has not been drawn thoroughly. One such approach has been made by Mellinger et al. (2007), who compared the performance distribution of six subjects performing an MI task with an MEG BCI with the performance distribution of a similar EEG study involving 96 subjects (Guger et al., 2003). However, the authors found no significant difference. In Bianchi et al.'s (2010) study data of 2 participants in MEG and another 4 participants in EEG have been compared at a single channel level regarding discrimination ability, revealing a more focused discriminative signal in MEG. The effect of spatial filtering has been investigated by Hill et al. (2006) who have found that MEG (6 subjects) does not perform better in MI classification compared to EEG (9 subjects), with EEG benefitting from spatial filtering but MEG not. The drawback of the previously mentioned studies is that they all compared different samples of brain activity. A more reliable approach would be the simultaneous EEG/MEG recording. One study which addresses this approach (Ahn et al., 2013) has shown that MI classification in MEG using the common spatial pattern (CSP) method as spatial filter is not significantly different from EEG. The authors argue that, according to Hill et al. (2006), the high number of channels overfit the spatial filter. In contrast, another spatial filter method based on beamforming performed better compared to features extracted from the sensor space in discriminating motor ERD (Battapady et al., 2009). However, a comparison to EEG has not been provided.

While BCI-related studies which compare EEG and MEG performance usually have investigated MI activity, occasionally combined with CSP, the detection of event-related potentials (ERP) and event-related fields (ERF) has rarely been targeted using simultaneous EEG/MEG. To our knowledge, just one study exists in this field, classifying single finger movements, with the finding that MEG is superior to EEG (Quandt et al., 2012).

In previous work, we have demonstrated BCI control based on ERF detection following a covert attention task (Reichert et al., 2013). Later we have shown that optimal spatial filtering considerably improves the accuracy in ERF detection (Reichert et al., 2015). Based on this approach, we developed an MEG based BCI, capable of discriminating 12 spatial locations on which covert attention has been shifted to by the users. The simultaneous recording of EEG and MEG permits a direct comparison between ERP and ERF signals regarding discriminability of temporally distinct sequences.

## Materials and methods

### Subjects

Nineteen subjects (ten female, mean age: 27.6 years SD = 4.1 years) participated in the study. All participants gave their written informed consent and had normal or corrected-to-normal vision. The study was approved by the ethics committee of the Medical Faculty of the Otto-von-Guericke University of Magdeburg.

### Recordings and task

The subjects were seated in a magnetically shielded room where MEG and EEG were simultaneously recorded while the subjects performed the experiment. The MEG data were streamed to a separate workstation to instantaneously process the data and provide feedback. Both MEG and EEG were sampled at 508.63 Hz with pass band from DC to 200 Hz and an amplitude resolution of 26.2 fT/bit and 118.51 nV/bit, respectively. MEG was recorded using a 248 channel magnetometer system (4D Neuroimaging Magnes 3600 WH) providing 23 additional reference sensors to capture environmental noise. The EEG was recorded at 29 standard positions (Fp1, Fp2, Fz, F3, F4, F7, F8, FC1, FC2, Cz, C3, C4, T7, T8, CP1, CP3, Pz, P3, P4, P7, P8, PO3, PO4, PO7, PO8, Oz, O9, O10, Iz) and referenced against the right mastoid. The impedance of the electrodes was kept below 5 kΩ. Furthermore, we monitored eye movements by recording the horizontal and vertical electrooculogram (EOG). EEG and EOG was recorded using a Sensorium EPA-6 amplifier.

**Figure 1:**
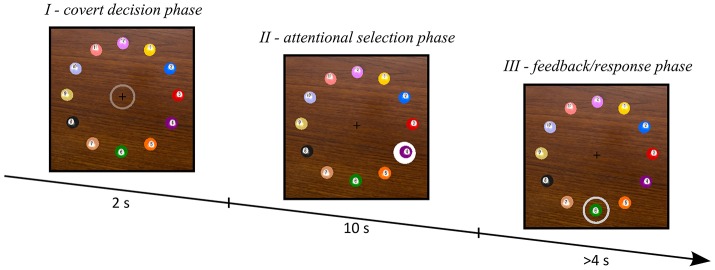
Depiction of the three phases of a trial. In phase *I* subjects made their decision, in phase *II* subjects selected their chosen target by attending the respective stimuli (the scene shows the moment when the 4th object was highlighted) and in phase *III* feedback was presented and the truly selected object was requested.

In a virtual scenario 12 colored and numbered (clockwise) spherical objects were presented throughout the whole experiment at equidistant places around a fixation cross (radius of 4.15° visual angle; see Figure 1). Each trial consisted of three phases. First, within 2 s subjects had to covertly choose one item as a target before each trial (*I-covert decision phase*). They were asked to select the items in an unpredictable order but try to balance the number of selections per item by selecting each object once per run. Second, to select an object, visual stimuli were provided while attention was directed to the target object (*II-attentional selection phase*). Subjects were asked to maintain gaze directed to the fixation cross but attend their chosen target covertly and ignore stimuli at all remaining locations. The stimulus at the attended target object could be considered an oddball, which elicits a characteristic brain response compared to the ignored stimuli (oddball paradigm). A decoding algorithm predicted which object location subjects directed their attention at. Each selection trial lasted 10 s while each object was highlighted with a temporally unique sequence of visual stimuli. More precisely, each of the 12 objects was highlighted (single flash of a white disk behind the object for 100 ms) five times in a pseudo-random order, resulting in 60 flashes in total with a stimulus onset asynchrony (SOA) of 167 ms. Minimum SOA between two successive stimuli of the same object was 500 ms, i.e., stimuli of any target object were separated by at least two non-target stimuli. In phase *III*, subjects were presented with the predicted object location and had to confirm or reject the choice of the computer (*III-feedback/response phase*). The object decoded by the detection algorithm was marked by a gray ring. The subjects had to respond whether or not the object was correctly recognized by pressing the thumb key for correct and the index finger key for incorrect feedback on a key pad. The feedback presentation in phase *III* was repeated until the correct item was found, where the item order was determined by the correlation measure generated by the detection algorithm using the MEG recordings of phase *II*.

In 40% of all trials we provided an incorrect feedback to evoke error potentials (Chavarriaga et al., 2014), which are typically measured when a subject perceives feedback different from his/her intention. In such cases, we did not present the object at rank 1 but we presented the second ranked object first. However, the analysis of error potentials is not part of the current paper. The duration of phase *III* depended on the number of incorrect feedback, and the time the subjects needed to respond (subjects were asked to respond clearly after the feedback presentation). The experiment was performed in runs of 12 trials each. The number of runs depended on the performance of subjects (10.7 runs on average).

### Data processing

#### Preprocessing

To decode the covert object selection in a single trial, we extracted a data segment ranging from the first stimulus onset in the sequence of visual stimuli to 800 ms after the last stimulus (total length of 10.63 s), adding 100 ms buffers for filtering and removing these samples afterwards. The MEG reference sensors were used to cancel environmental noise from MEG channels as proposed by Robinson (1989). We excluded three MEG sensors from the analysis due to malfunction. To reduce the amount of data and decrease calculation time, we applied a low-pass FIR filter at the target Nyquist frequency and down-sampled the data segment by factor 10, resulting in a sampling frequency of 50.863 Hz.

#### Modeling reference functions

Our algorithm for detecting ERPs/ERFs is based on the idea of matching the brain signals with a set of reference signals which model the time course of the brain response to the target stimuli. Commonly, those template-matching approaches are applied to single channels, using a hypothetically defined template signal (Fabiani et al., 1987; Smulders et al., 1994). Here, we make no assumptions on the location or the time course of the signal. Instead, we determine a set of optimal template signals by means of statistical learning theory from multichannel brain signals, which we call *matched filters*.

The pre-processed brain signals of one trial represent a data matrix ***X*** ∈ ℝ^*n*×*c*^ consisting of *n* sampling points by *c* channels. According to our stimulation scheme, within the 10 s of the trial 60 flashes were presented with 5 flashes at each of the 12 locations. For example, the object at one location was highlighted with flash no. (12, 20, 27, 38, 59) while the object at another location was highlighted with flash no. (2, 19, 30, 37, 56). Hence, each location is defined by a unique temporal signature of flashes, i.e., a unique stimulus sequence.

Depending on a trial's stimulus sequences, we modeled a set of reference functions for each of the *m* locations. Similar to the method in Reichert et al. (2015) the reference functions were defined as a set of *d* impulse functions for each stimulus sequence specific to an object location *e* = 1 …*m*, where the *j*th sample point after stimulus onset (*j* = 1 …*d*) is set to 1, and 0 otherwise. More precisely, we define sets of reference functions ${Y_{e}\in \mathbb{R}}^{n\times d}$, where one element in ***Y***_*e*_ (at sample point *i* and reference function *j*) is defined as

(1) $$\begin{array}{l} y_{e}\left(i,j\right)=\{\begin{array}{c} 1|i=j+t,\forall t\in t_{e.}\\ 0\;otherwise \end{array} \end{array}$$

The parameter *t* constitutes the sample point at which one of the stimulus onsets in ***t***_*e*_ of object location *e* occurred. In this study, we used *d* = 41 sample points, which corresponds to a time window of 0.8 s. Note that this kind of function set can be seen as a matrix which maps an arbitrary matched filter ***s*** ∈ ℝ^*d*×1^ into a sequence of repetitive copies of ***s***, starting at onsets ***t***_*e*_, by ordinary matrix multiplication:

(2) $$\begin{array}{l} \begin{array}{c} v_{e}\;=\;Y_{e}s. \end{array} \end{array}$$

**Figure 2:**
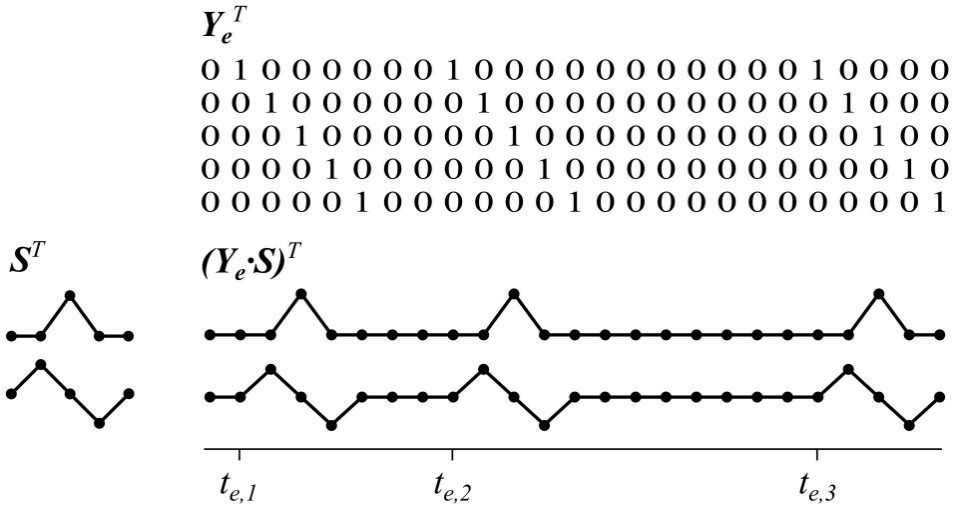
Reference functions which copy intervals of short signals according to a sequence of event onsets. For demonstration purposes only three event onsets, a time interval of five sample points and two sample signals are shown. Matrices are depicted transposed.

We assume that all target events elicit comparable brain responses but no systematic activity at non-target stimuli. Then, ***v***_*e*_ can be considered a kind of event-related signal component that models the evolution of ***s*** following each target stimulus. See Figure 2 for a visualization of these relationships.

The use of one impulse function for each time point in ***s*** enables us to estimate the matched filter as an arbitrary signal time course with a minimum of orthogonal functions, which is in contrast to the approach introduced by Spüler et al. (2014b) who applied the average signals of each channel as reference signals. Next we explain how we determined the matched filters ***s***, which are assumed to optimally represent the measured brain signals.

#### CCA spatial filtering

A method capable of solving this problem is the canonical correlation analysis (CCA), which maximizes the correlation between linear combinations of two sets of variables. Applied to the present problem, CCA determines a set of spatial filters that linearly weight channels of recorded brain signals to enhance the signal strength and simultaneously determines a set of matched filters that weight the signal at time points after target event onset such that the correlation ρ_*k*_(***u***_*k*_, ***v***_*k*_) of the *k*th component is maximal, where

(3) $$\begin{array}{l} u_{k}\;=\;X_{Wk} \end{array}$$

and

(4) $$\begin{array}{l} v_{k}\;=\;Ys_{k}. \end{array}$$

**Figure 3:**
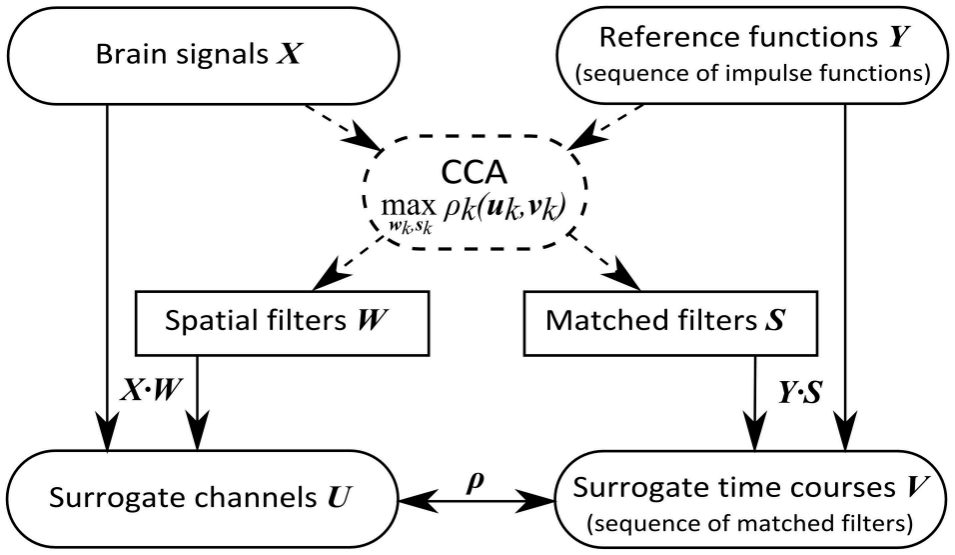
Concept of the optimal spatial filtering approach. While the CCA is only applied to training data (dashed lines), for the filtering of new data (solid lines) ordinary matrix multiplication is sufficient.

Consequently, CCA reveals a vector ***w***_*k*_ of channel weights, which we call *spatial filter*, transforming brain signals ***X*** into the canonical variate ***u***_*k*_, which can be seen as *surrogate channel*. Simultaneously, CCA reveals a vector ***s***_*k*_ of weights, representing a signal template following target events, termed *matched filter*. The canonical variate ***v***_*k*_ can be considered a *surrogate time course* reflecting the potential event-related components of a stimulation sequence. Similar to the principal component analysis (PCA), the optimization criterion decreases with increasing *k*, rendering the first components most relevant. In Figure 3 we provide an overview of our optimal spatial filter concept.

We performed the estimation of the linear weightings, applying CCA, by using a set of training trials which we concatenated along the time domain to a common matrix ***X*** and a common matrix ***Y***. Since the correlation decreases with increasing *k*, we obtain the surrogate channels sorted by significance in descending order. To reduce the number of surrogate channels for further processing, we removed canonical variates providing canonical correlations ρ_*k*_(***u***_*k*_, ***v***_*k*_) below 0.1 or providing a zero correlation probability value below 0.05, which was obtained from a χ^2^ statistic for the null hypothesis that all canonical correlations ρ_*i*_(***u***_*i*_, ***v***_*i*_), ∀*i* ≥ *k* are zero.

#### Decoding algorithm

Using brain activity, the decoding algorithm makes predictions to which position the subjects shift attention, based on identifying the event sequence specific to the object location. After the optimal filters were estimated by CCA from a set of training data, they can be used to transform new data to surrogate channels, representing a canonical subspace. For each trial the stimulus sequence for each object *e* is assigned in ***Y***_*e*_. The surrogate channels ***u***_*k*_ are calculated with Equation (3) and surrogate time courses ***v***_*k,e*_ are calculated for each object location with Equation (4). Afterwards, for each object the correlation ρ_*k,e*_(***u***_*k*_, ***v***_*k,e*_) is calculated. Subsequently, the correlations are transformed applying the inverse hyperbolic tangent and averaged across components. This reveals a vector of *m* average correlations, serving as a ranking measure for the probability that an object was attended by the subject, the highest correlation denoting the most probable object.

### Validation

The previously described decoding approach was applied during the experiment, providing the participants a feedback, attention to which of the items the decoder recognized from their brain activity. In the first trial, the spatial filter ***w*** was initialized with *w*_*i*_ = 1, *i* = 1 …*c*, resulting in an average filter across all channels and ***s*** was initialized as hat function (triangular function), constituting a simplified ERP signal. This enabled us to make a prediction even in the first trial. Subsequently, ***w***_*k*_ and ***s***_*k*_ were determined using the CCA method involving all available training data, i.e., from previous trials. In the initial two runs the filter estimation was performed after each trial, afterwards after each run.

We report the decoding accuracy (DA) as the ratio of correctly decoded target objects and total number of trials. Despite having serious limitations, the information transfer rate (ITR) according to Wolpaw et al. (1998) is commonly used to compare BCI performance, taking DA, trial length and number of selectable items into account. Here we report practical ITRs, i.e., an interval for feedback presentation and preparation for the next trial is included, which we assume to be 2.5 s, in addition to the stimulus duration.

### Post-analyses

#### Suitability of MEG and EEG for decoding ERPs

Given the low amount of individual training data, we applied a leave-one-run-out cross-validation framework to compare the MEG data with the EEG data using the same algorithms and data acquired during the same trials for training and testing, respectively.

Although the proposed method is suitable to be applied to full channel sets, we additionally investigated subsets of channels to compare EEG and MEG with a matched number of channels. We selected the channels of a subset according to their contribution to canonical variates of brain signals. Therefore, we first applied CCA to the whole channel array and then repeated CCA with the channel subset of size N, selected by the highest weightings in ***w*** from the initial CCA. Note that finding the *N* most contributing channels reveals the potential power of either modality.

The spatial filter we used here is a data-driven method, which makes no assumptions as to the location or scaling of a channel. Furthermore, because electrical and magnetic fluctuations are perfectly synchronized, an appropriate combination of the channels of either modality might complement each other to a more representative signal component. Therefore, we combined both signal modalities by providing the algorithm with MEG and EEG channel data in parallel and rely on the data-driven spatial filter approach to find the most predictive event-related component. We also tested the influence of stimulation duration on the DA in order to investigate the performance of faster BCI control. To achieve this, we truncated the analysis intervals to lengths of 2, 4, 6, and 8s, which corresponds to 1, 2, 3, and 4 stimuli per item.

#### Transfer learning

With the aim to reduce training time, we investigated the ability of transfer learning with either modality (MEG/EEG) by performing a leave-one-subject-out cross-validation, i.e., each trial is decoded by a classifier that was trained on all available data of subjects except the individual.

Furthermore, we investigated the dependence of DA on the amount of training data by using a maximum number of chronologically recorded trials to construct the decoder. Moreover, we entered data from other subjects and used this pool of data as an initial training set to provide a classifier for the actual subject, which subsequently is incrementally substituted by individual data. As initial data pool we used 100 trials of group recordings, providing the highest canonical correlation revealed in the spatial filter construction using all available trials but not those from the current individual, assuming that these are the most reliable trials for generalization. Here we used only a subset of the available trials to enable the decoder construction in a time suitable for BCI application on the one hand, and to be able to completely replace the group trials by individual trials on the other hand.

#### Statistical evaluation

To make sure that the decoding procedure reveals reasonable results, we determined the guessing level of the classifier by performing a permutation test. For this purpose we randomly reassigned the labels of the object-specific sequences and performed the cross-validation approach for each subject. This implies the assumption that subjects directed their attention to a randomly selected object which they actually not attended. We repeated the permutation test 500 times for each subject providing a joint distribution of decoding accuracies. The guessing level is calculated by the mean value of this distribution and confidence intervals are given by determining the 95% percentile, respectively.

For comparison of DA between any kinds of two methods we applied paired, two-sided Wilcoxon signed rank tests. We applied non-parametric test statistics due to the low number of samples (one observation per subject). A paired signed test was chosen, because we were interested in the individual change of a relatively wide distributed performance measure.

Different combinations of training data, like combining EEG and MEG compared to EEG or MEG only could reveal different filters. In order to investigate the robustness of the spatial filter approach, we compared its outcome by calculating Pearson's correlation coefficients *r* between the obtained components. Since *r* values are not a metric parameter, individual *r* values were transformed by applying the inverse hyperbolic tangent before statistical analyses.

## Results

### Control for eye movements

We monitored eye movements sporadically during the experiment on a video screen and systematically offline by means of EOG recording. The EOG data showed no evidence that subjects moved their eyes to different locations. Eye blinks occurred mainly outside the attentional selection phase. We performed a cross validation applying the proposed algorithm but using the two EOG channels instead of EEG or MEG. The EOG activity was not sensitive as to the target stimuli (average DA was 19.3%; SD: 6.4%) as EEG or MEG activity (see below).

### Validation of decoding accuracy

Subjects freely selected the objects starting with the first trial, i.e., without a cue-based training phase. They followed the instruction to select targets equally often with only small deviations (average standard error for selecting each object once per run, i.e., 10.7 times, was 0.18). After each trial they responded with a button press whether or not the feedback indicated the correct selection. This notification provided the information needed to update the spatial filter and the decoder, respectively. Using only MEG data in this closed-loop BCI, the average DA across subjects was 91.1% (SD: 7.6%). This corresponds to a practical ITR of 13.9 bit/min (SD: 2.3 bit/min), assuming a trial length of 12.5 s.

**Figure 4:**
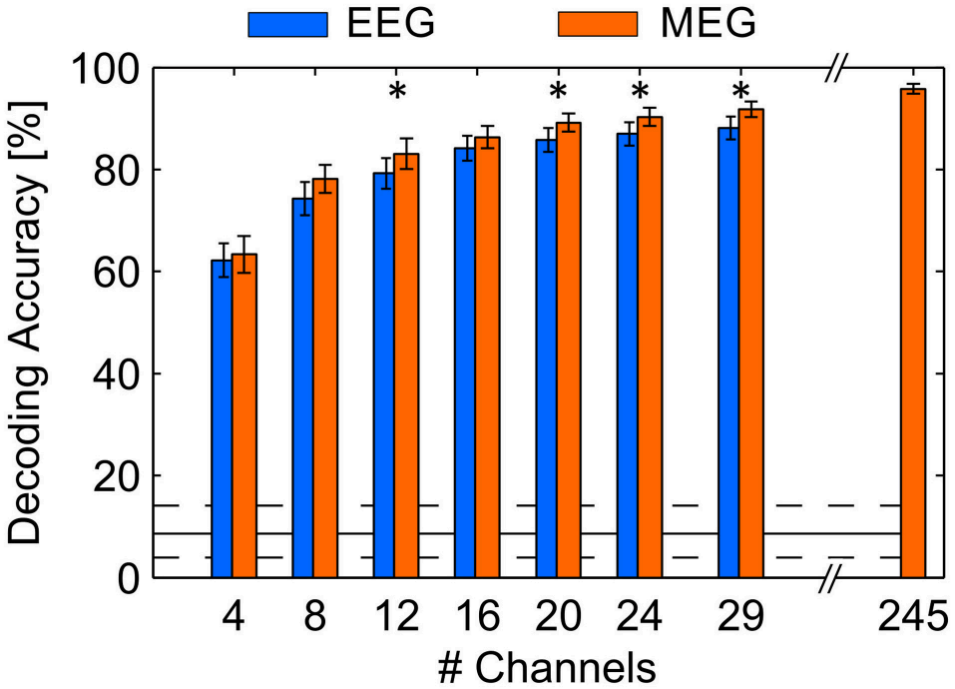
Average DA achieved in a cross-validation procedure using EEG and MEG involving different numbers of channels. The maximum number of EEG channels was 29, the maximum number of MEG channels was 245. Error bars indicate standard error of the mean, asterisks indicate *p* < 0.05. The solid black line indicates the guessing level determined as mean DA in a permutation test, where the dashed lines mark the 95% confidence interval.

In order to compare the decoding method for the use with MEG signals and the use with EEG signals, we performed cross-validations, leaving one run out (12 trials) as a testing set in each cycle. This approach provides a constantly high number of training samples to the learning algorithm, contrary to predicting in an online fashion, where only past data are considered. Hence, the cross validation approach revealed an average DA of 95.8% (SD: 4.2%) using the full MEG sensor array. In contrast, average DA achieved with the simultaneously recorded EEG signals was 88.2% (SD: 9.7%) indicating a significantly higher DA with MEG (*p* < 0.05). However, because MEG has the advantage of providing a higher number of spatial locations where signals are measured, the decoding approach might benefit from a higher information content. Therefore, we tested both modalities depending on the number of channels involved, where only the *N* channels (*N* = 4, 8, 12, 16, 20, 24, 29) revealing the highest spatial filter weightings, obtained by CCA on the full channel array, were selected within each cross-validation cycle. We found that DA increased as a function of the number of channels (see Figure 4). MEG benefits from the higher number of sensors, since DA was significantly different using 29 MEG sensors (90.3%, SD: 6.7%) compared to using all available sensors (*p* < 0.05). However, compared to EEG, the MEG recordings also permitted higher DA keeping the number of involved channels equal for both modalities (*p* < 0.05 with *N* = 12, 20, 24, 29). Also, an analysis of variance (ANOVA) using factors modality [*F*_(6, 252)_ = 4.74, *p* < 0.05] and number of channels [*F*_(6, 252)_ = 26.68, *p* < 0.05] revealed statistically significant differences. Note that in this analyses the selection of channels in MEG might be beneficial due to the higher number of candidates.

**Figure 5:**
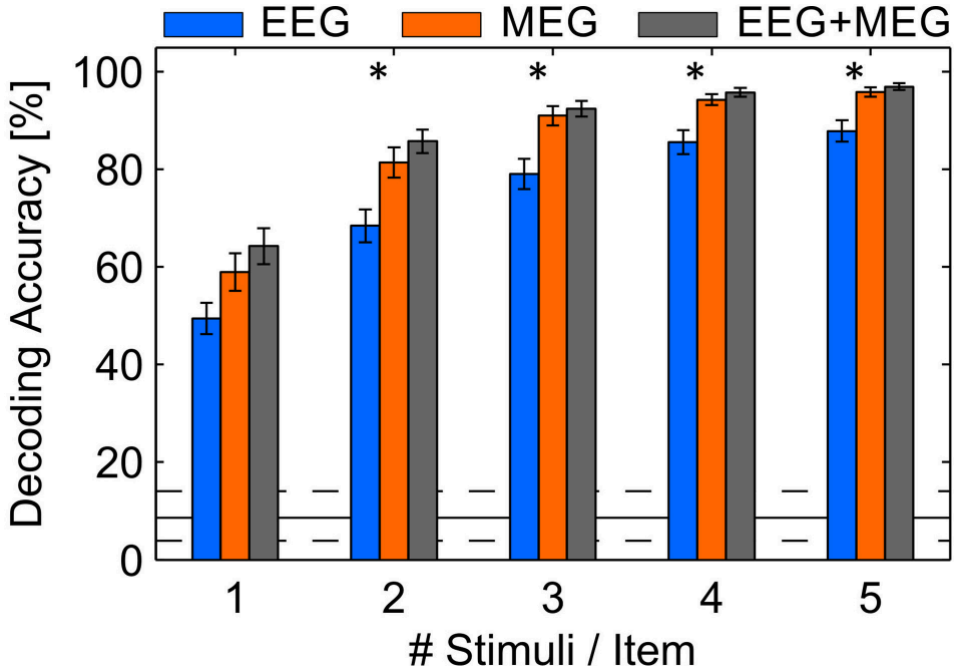
Average DA achieved in a cross-validation using EEG, MEG and combined EEG and MEG involving different numbers of stimuli per item. Error bars indicate standard error of the mean, asterisks indicate *p* < 0.05. The solid black line indicates the guessing level determined as mean DA in a permutation test, where the dashed lines mark the 95% confidence interval.

The spatial filter approach we used is unspecific to the location of channels, suggesting that it is also possible to merge EEG and MEG signals. Because it is assumed that EEG and MEG provide mutually complementing signals (Hämäläinen et al., 1993), we tested whether EEG provides additional information. Hence, we merged the EEG and MEG channels into one data set, assuming each of the channels include some subcomponent of the underlying brain processes. We found a statistically significant increase of the DA to 97.0% (SD: 3.2%; *p* < 0.05). Because this result is quite close to perfect prediction, one might argue that there is few potential for improvements. Thus, we truncated the stimuli sequences which induces a decrease of decoding performance but would permit a higher communication speed (see Figure 5). The shortest sequence is composed of one stimulus per item and trial which would result in a stimulus duration of 2 s. In each of the sequence lengths (2, 4, …, 10 s) the MEG permits a higher DA than EEG and combined EEG/MEG is even higher. An ANOVA with factors modality and number of stimuli proved statistically significant difference between modalities [*F*_(8, 270)_ = 36.6, *p* < 0.05] and stimulation time [*F*_(8, 270)_ = 102.2, *p* < 0.05] but no interaction effect. Although the gain with combined modalities is significant (*p* < 0.05), the numerical difference is relatively low. However, the relative decrease of MEG performance with shorter trials is significantly lower compared to the relative decrease in EEG performance using 2 and 3 stimuli per item (*p* < 0.05). Importantly, the results reveal that 3 stimuli per item (6 s of stimulation) are sufficient to decode the attended item with more than 90% accuracy using MEG or combined EEG/MEG. Thus, assuming a trial length of 8.5 for 3 repetitions per item, an average ITR of 19.6 bit/min could be achieved. In contrast, EEG shows an average accuracy of 79.1% (SD: 13.5%) implying 15.0 bit/min.

### Transfer learning

**Figure 6:**
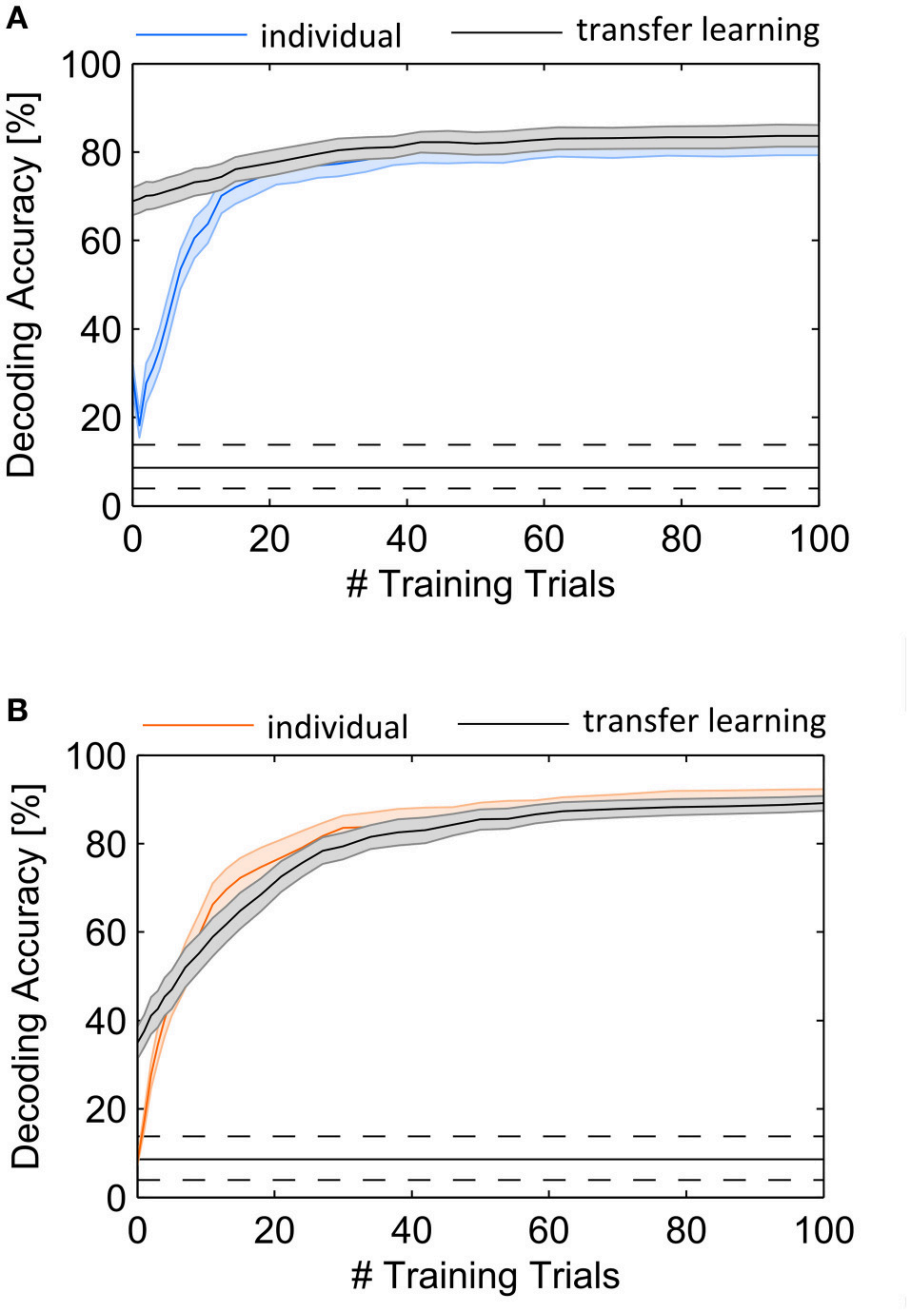
Average DA as a function of the number of training trials used to estimate the spatial filter. The performance rapidly increases with the first 20 trials using only the individual EEG **(A)** and MEG **(B)** recordings. Involvement of cross-subject trials (black lines) permits high performance with the first trial in EEG but not in MEG. Shaded areas indicate standard error of the mean.

In this experiment we predicted attended objects using individual spatial filters which were updated during the course of the experiment, where accuracy increased with the number of trials. Hence, a critical drawback in all BCI applications is the amount of training needed to achieve reliable performance of the system. In this regard a critical question is whether information of other users can help to accelerate or even avoid the training process. First, we investigated the development of DA in subjects individually, by iteratively increasing the set of training trials. This gives a DA for each set of 1 to *N* trials. The DA dependence on the amount of individual training is shown in Figure 6. Our first observation is that the initial spatial filter, which generates the mean signal over all sensors only from the very first trial, achieves poor performance in EEG as well as in MEG. However, in contrast to MEG the performance of the initial spatial filter in EEG is above guessing level and higher than applying a trained spatial filter using only one training trial (indicated by the trough in Figure 6A). The figure also reveals a continuous increase of DA with increasing number of training samples both in EEG and MEG. We determined the number of trials that are sufficient to achieve a DA not significantly different from using 100 training trials, where DA was 81.9% (SD: 11.6%) with EEG and 90.5% (SD: 8.2%) with MEG, indicated by *p* < 0.05. As a result, 62 training trials for EEG and 94 for MEG were found using a signed rank test. Note that the cross-validation results reported in the previous section provide higher accuracies because each trial is tested with a well-trained spatial filter estimated from up to 120 trials (each involving 5 target stimuli). In the current validation scheme, simulating an online prediction, only past data are used and hence, early trials are decoded from a sparsely trained spatial filter. This kind of simulation permits the validation of EEG for using our approach online. The average accuracy achieved after 100 trials accords to a practical ITR of 11.2 bit/min (SD: 3.0 bit/min) when assuming a trial length of 12.5 s.

In a second analysis we tested the ability of transfer learning by initially using a pool of 100 training trials from different subjects and iteratively replacing trials with individual subject data until the training set consisted of pure individual data. The result of this analysis is shown with the black lines in Figure 6. The selection of trials from different subjects revealed an initial accuracy of 68.9% (SD: 13.6%) for EEG and 34.9% (SD: 16.0%) for MEG, where no data of the current subject were required. With successive inclusion of individual subject data for the spatial filter estimation the DA increases, approaching the level of individual subject performance. This approach reveals a higher benefit of EEG with the cross-subject approach, specifically for the first trials. An ANOVA revealed significant differences between DA in individual decoder training and transfer learning for the first 9 trials in EEG and for the first 2 trials in MEG (*p* < 0.05).

Moreover, transfer learning across subjects was tested here in a leave-one-subject out cross validation, using all available trials from different subjects. This yielded a general spatial filter which we used to predict the attended item of the left-out subject. The approach provides a performance estimate for the case that a new subject would use the BCI without any training. Here we found a statistically significant better performance on EEG (mean DA 71.0%, SD: 13.9%) compared to MEG data (55.0%, SD: 16.0%; *p* < 0.05). This suggests that EEG data provide better generalization across subjects compared to MEG. However, the successful application of a generalized spatial filter depends on the subject, which is implied by strongly varying decoding accuracies achieved with single subjects, ranging from 36.2 to 94.7% using EEG signals. The performance of subjects in within-subject cross-validation and in the across-subject analysis is strongly correlated (*r* = 0.9).

### Evaluation of extracted brain signals

**Figure 7:**
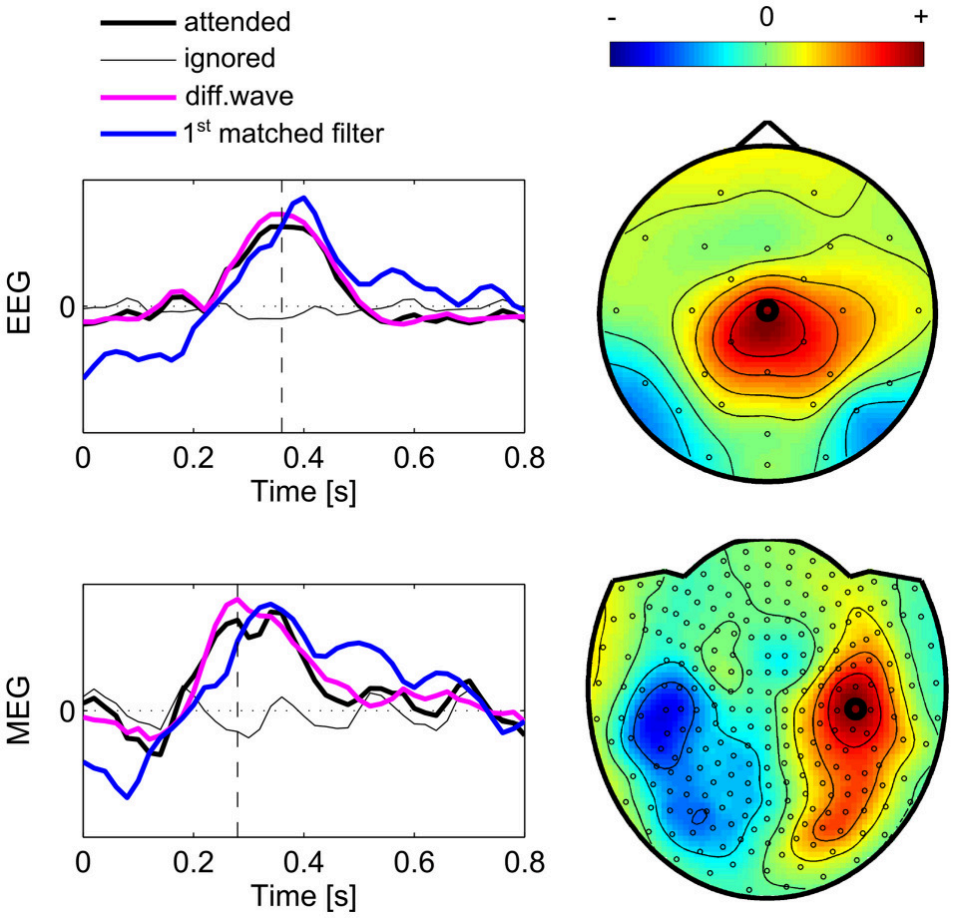
Subject group average of brain signals. The diagrams show average signal courses of the sensor (marked in the topographic maps) showing the highest difference between signals following attended and ignored stimuli. Black lines represent averaged signals over intervals following ignored stimuli (thin line) and attended stimuli (thick line). The difference wave indicates the attentional component which shows a characteristic P300. The peak of this difference waves defines the time point (vertical dashed line) which is shown with the topographic maps. The first matched filter (blue line) shows a time course similar to the averaged signal of the channel peaking in the “attended” condition. Due to scaling, units are arbitrary.

The matched filters as revealed by CCA denote optimal templates of the brain responses to attended stimuli. Here we evaluated the first matched filter ***s***_1_ across subjects indicating the most significant event-related component in relation to the average signal (Figure 7). The figure also shows the topographic maps at the peak signal of the difference waves, which were calculated as the difference between the mean signals acquired during attended events and ignored events. The diagrams indicate the event-related brain signal at the channel showing the highest peak in the difference wave. Here, the average signal of both conditions “attended” and “ignored” are shown as well as their difference and the highest ranked matched filter. The first observation is that in the “ignored” condition, as well as in the “attended” condition the signal is modulated by an oscillation around 6 Hz. This common signal fluctuation corresponds to the stimulation frequency (every 167 ms one of the items is highlighted) and is canceled in the difference wave but also present in the matched filter. Thus, this oscillation most likely reflects brain activity, which is evoked after each of the visual stimuli, implicated by brain processes following a visual stimulus. Also, because the stimulation frequency is approximately at 0.5 times the *alpha* frequency, resonance phenomena could be involved in this effect (Salchow et al., 2016). However, because these visually evoked ERPs are present in both target and non-target trials, they have no impact on the detection of the target. The first matched filter ***s***_1_ in EEG data reveals a component showing a positive peak around 400 ms, where the maximum signal is located at Cz, concordant with the signals measured in oddball paradigms, where it is established as the P300 response. While the signal course of the first matched filter in MEG is similar, but showing the peak earlier (around 340 ms) than EEG, the channels which show the maximum signal are located bilaterally over left-central and right-central regions. The patterns reflect the typical orthogonality of effects measured with EEG and MEG. In both modalities the estimated matched filter appears to be delayed by 40–60 ms compared to the difference waves of the channels showing the highest peak.

Note that the spatial filters and matched filters found in single subjects differ in spatial distribution, shape and ranking. Given that the first component provides the highest contribution to the detection of the target event, we restricted the single subject evaluation to this component. Thus, we compared the matched filters obtained in single subjects with the first matched filter of the whole dataset by determining the correlation coefficient and selecting the filter with highest similarity but a correlation of at least $\bar{\rho }$ > 0.5. For the EEG data in 14 subjects the first matched filter of the individual was highest correlated with the first matched filter of the group and in four subjects the secondly ranked component of the individual was highest correlated with the first group component ($\bar{\rho }=0.837$). For the MEG data, the first matched filter was equally ranked for all subjects where the average correlation was $\bar{\rho }=0.909$.

Finally, we evaluated the first matched filter extracted in single subject analysis with EEG data, MEG data, and combined EEG/MEG data. The first matched filter obtained with combined EEG/MEG correlated using only EEG signals with $\bar{\rho }=0.932$. When using only MEG data the correlation was $\bar{\rho }=0.989$, suggesting a higher impact of MEG data in the combined data set. Also, comparing the spatial filter vectors ***w***_1_, the average correlation is high for the spatial filter in the EEG data set and the channel weights corresponding to EEG in the combined data set ($\bar{\rho }=0.902$). Again, for MEG channels this correlation is even higher ($\bar{\rho }=0.975$).

## Discussion

We implemented a BCI system based on the decoding of event-related magnetic fields evoked by covert attention. Simultaneous EEG recordings were analyzed with the same data-driven decoding algorithm to compare the performance of ERP and ERF decoding. We found that ERFs can be more accurately decoded than ERPs using single subject cross validation. However, the transfer of learned brain patterns between subjects was better with EEG. The method used here proved to be a suitable algorithm to extract informative subcomponents from sensor data that permit a reliable prediction of spatial attention.

We applied a data-driven spatial filter that learned optimal linear combination of channels from a set of training data. Correlation of the linearly combined channels with the estimated optimal reference signal indicated the degree of similarity of brain responses to different stimulus sequences. With this approach we could predict the covertly attended object with high accuracy. The circular arrangement of objects guaranteed an equidistant location of all objects around the fixation cross. Furthermore, the classifier decoded sequences of events that were unspecific to locations. Thus, it is unlikely that brain responses evoked in the visual system, focally or peripherally stimulated, contribute to ERP/ERF decoding. Previous work has revealed that visual brain activity biases the prediction in matrix spellers when subjects focus on stimuli (Brunner et al., 2010), but the potential users of such a system might not be able to shift their gaze. The number of studies considering this requirement in their communication systems is small compared to the amount of gaze-dependent BCI studies. In a review article (Riccio et al., 2012) 34 papers dealing with gaze independent BCIs were reviewed. The average ITR of the reported studies was in auditory BCIs 3.5 bit/min (SD: 2.8 bit/min), in tactile BCIs 2.6 bit/min (SD: 1.4 bit/min) and in independent visual BCIs 6.1 bit/min (SD: 2.8 bit/min). Promising transfer rates were reported from offline analyses investigating independent BCIs achieving theoretical rates of 10.5 bit/min (Acqualagna and Blankertz, 2013). Recently, it has been shown that colored facial expression stimuli can induce an average online ITR of 13.9 bit/min (Chen et al., 2016). Another study reported an average ITR of 10.77 bit/min using a bimodal auditory-tactile BCI (Yin et al., 2016). The online ITR of 13.9 bit/min achieved in our study is comparable or even exceeds the-ITR of other approaches targeting gaze independence. This shows that the decoding approach is well-suited for detecting sequences of ERFs elicited after an attended target was highlighted. Additionally, in offline analyses we showed that the ERP decoding with our method also revealed an ITR above 11 bit/min.

A key feature of our approach is the data-driven estimation of spatial filters, which permits the application of the algorithm to EEG and MEG data and a combination of both, without hypothetically determining relevant channels. Comparing EEG with MEG showed that decoding ERFs reveals superior accuracy as compared to ERP decoding. This supports the findings of Quandt et al. (2012), who could discriminate individual finger movements on one hand significantly better with MEG. Given that motor potentials are elicited in a small region during finger movement execution, this result is most likely due to a higher spatial resolution of MEG. The advantageous spatial resolution of MEG results from the absence of distortions of magnetic fields by tissues, which is in contrast to EEG where the electrical field is spread by volume conduction (Wheless et al., 2004). However, the spatial resolution of MEG also relies on the higher number of channels as shown by the higher accuracy achieved using the full sensor array compared to a sensor set reduced to the number of EEG electrodes. Nevertheless, when we used equal numbers of channels in both modalities, MEG proved to be significantly better than EEG. In contrast, the blurring effect caused by volume conduction in EEG, most likely rules out an improved DA with only a higher number of electrodes. The question whether or not high density EEG improves DA should be addressed in future investigations.

While our results contribute to clarify the controversies on methodological advantages of MEG, we are aware that this technique implicates limitations for BCI use. The lack of mobility and the sensitivity to magnetic fields induced by electrical currents and ferromagnetic material is a limitation especially for controlling robotic devices. Despite the superior DA, MEG is associated with more practical cost and might be considered less feasible, although upcoming new MEG technologies (Alem et al., 2017) may allow for better practicability. Nevertheless, a use for occasional communication sessions in severely paralyzed persons is conceivable. Also, the rehabilitation of stroke patients could be a field of application (Silvoni et al., 2011).

A further statistically significant increase of DA could be observed when we provided combined EEG/MEG to the spatial filter algorithm. This supports the notion that EEG and MEG are complementary methods because EEG mainly measures radially oriented currents while MEG can only measure tangentially oriented currents (Hämäläinen et al., 1993). The advantage of combined bioelectric and biomagnetic data has also been shown in reconstructing source dipoles (Fuchs et al., 1998; Baillet et al., 1999; Sharon et al., 2007) and coherence-based network analysis (Muthuraman et al., 2014). When we used the two modalities combined in our study, MEG determined more strongly the time course of the components obtained by the spatial filter. Furthermore, the matched filter in MEG correlated higher than in EEG with the filter obtained from combined data, corroborating the superiority of MEG.

We also investigated whether the advantage of MEG and combined EEG/MEG is higher with shorter trial lengths. As expected, the DA decreased with shorter trial lengths but the relative decrease was lower in MEG (compared to EEG using 40% less stimuli), still achieving more than 90% accuracy on average and permitting a higher ITR compared to the initial trial length. The results suggest that MEG and combined EEG/MEG is suitable for a fast and accurate selection of one out of 12 items within 6 s. The gain of DA in combined EEG/MEG is significant but presumably too low for the effort of additional EEG measurements.

A known issue in BCI control is the requirement of training and calibration for a new user and in most cases even for every session. Therefore, algorithms and techniques permitting transfer learning from group data applicable to individual subjects are of high interest. We found that a relatively high DA can be achieved with EEG, where some subjects match the group pattern much better than others. In contrast, the transferability of an MEG based decoder is much worse where accuracy is insufficient for communication. One reason for the limited transfer performance of MEG could be that the subject's head is individually positioned under the sensor array while EEG channels have standardized positions. Thus, channel weightings of narrow local activity can easily be shifted to other channels between subjects in MEG. Another reason could be the higher diversity of MEG patterns, which are sensitive to the individual location and orientation of dipoles induced by intracellular currents as opposed to the EEG patterns generated by volume currents. Potentially, a source reconstruction could reveal better transfer learning results when classifying MEG data. Importantly, the successful application of group data to predict covert attention in a new subject as in our study is an important aspect to reduce calibration time and hence to improve the usage of BCIs.

Further validations revealed that when updating the cross-subject decoder with individual data, accuracy increased to converge with results of pure individual data. Certainly, adding individual data for decoder training requires the true label of a trial which is only available in a user training mode but this analysis gives an estimate of the accuracy achievable with a specific amount of training. The time required for training with our approach is quite short compared to other approaches and could even be omitted using EEG as suggested by the cross-subject analysis.

The strength of our approach is that spatial filters and matched filters are estimated simultaneously without any hypothesis about time course and location of the signals. A comparable approach aiming at reducing MEG channel signals representing an experimental effect in a single time course was introduced in Schurger et al. (2013). However, while in this approach average signals are projected in each time step, a simultaneous optimization of spatial and temporal filters is not provided. A method strongly related to spatial filtering and often used to estimate dipole locations is beamforming. Related to our approach it is important to note that in contrast to beamforming, the weights we obtain are independent of any spatial information. This implies that sources cannot be localized and that the components still might be composed of different superimposed sources. Rather, the linear combinations maximize the correlation with a model of the target signal, being most relevant for the detection of ERPs/ERFs. A data-driven beamforming approach, which similarly to our approach is not able to localize sources but is intended to detect single-trial ERPs, was already introduced by Treder et al. (2016). The advantage of spatial filtering using CCA for ERP detection has been demonstrated using EEG (Spüler et al., 2014b) where trial averages were used as reference functions. Here we used an approach similar to Reichert et al. (2015), estimating the reference signal from the brain signals. The most prominent signal component showed a time course which is typical for oddball paradigms producing the P300 response. Also, the variability among individual brain patterns reveals further signal components which vary among subjects in shape and rank of their canonical correlation. Nevertheless, the components extracted in single subjects were highly correlated, demonstrating the plausibility of the approach. The reliability of CCA for extracting task-related signal components we also demonstrated by the similarity of components and spatial filters obtained from EEG, MEG and combined EEG/MEG signals.

An investigation of P300 components using spatial PCA has been published by Spencer et al. (2001). The methods used in this study have much in common with the CCA spatial filter. While the authors of this study evaluate the decomposition of virtual ERPs, they make no use of the extracted components to decode attended and ignored events. In this regard, to our knowledge this is the first study which performs single trial detection of ERFs by applying sophisticated signal processing and compares the result with concurrently evoked ERPs as well as with a multi-modal approach which combines both, highlighting an advantage of MEG over EEG.

## Author contributions

CR: study design, BCI implementation, data acquisition, and data analysis; CR and SD: drafting of manuscript; CR, SD, HJH, and HH: data interpretation and manuscript revision. All authors agree to be accountable for the content of the work.

### Conflict of interest statement

The authors declare that the research was conducted in the absence of any commercial or financial relationships that could be construed as a potential conflict of interest.
